# Cannabidiol Promotes Endothelial Cell Survival by Heme Oxygenase-1-Mediated Autophagy

**DOI:** 10.3390/cells9071703

**Published:** 2020-07-16

**Authors:** Sabine Böckmann, Burkhard Hinz

**Affiliations:** Institute of Pharmacology and Toxicology, Rostock University Medical Center, Schillingallee 70, D-18057 Rostock, Germany; sabine.boeckmann@med.uni-rostock.de

**Keywords:** cannabidiol, heme oxygenase-1, endothelial cells, apoptosis, autophagy

## Abstract

Cannabidiol (CBD), a non-psychoactive cannabinoid, has been reported to mediate antioxidant, anti-inflammatory, and anti-angiogenic effects in endothelial cells. This study investigated the influence of CBD on the expression of heme oxygenase-1 (HO-1) and its functional role in regulating metabolic, autophagic, and apoptotic processes of human umbilical vein endothelial cells (HUVEC). Concentrations up to 10 µM CBD showed a concentration-dependent increase of HO-1 mRNA and protein and an increase of the HO-1-regulating transcription factor nuclear factor erythroid 2-related factor 2 (Nrf2). CBD-induced HO-1 expression was not decreased by antagonists of cannabinoid-activated receptors (CB_1_, CB_2_, transient receptor potential vanilloid 1), but by the reactive oxygen species (ROS) scavenger N-acetyl-L-cysteine (NAC). The incubation of HUVEC with 6 µM CBD resulted in increased metabolic activity, while 10 µM CBD caused decreased metabolic activity and an induction of apoptosis, as demonstrated by enhanced caspase-3 cleavage. In addition, CBD triggered a concentration-dependent increase of the autophagy marker LC3A/B-II. Both CBD-induced LC3A/B-II levels and caspase-3 cleavage were reduced by NAC. The inhibition of autophagy by bafilomycin A_1_ led to apoptosis induction by 6 µM CBD and a further increase of the proapoptotic effect of 10 µM CBD. On the other hand, the inhibition of HO-1 activity with tin protoporphyrin IX (SnPPIX) or knockdown of HO-1 expression by Nrf2 siRNA was associated with a decrease in CBD-mediated autophagy and apoptosis. In summary, our data show for the first time ROS-mediated HO-1 expression in endothelial cells as a mechanism by which CBD mediates protective autophagy, which at higher CBD concentrations, however, can no longer prevent cell death inducing apoptosis.

## 1. Introduction

Atherogenesis is the main cause of pathological cardiovascular events such as heart disease and stroke [1]. Within this multifactorial process, endothelial dysfunction, neovascularization, vascular proliferation, apoptosis, matrix degradation, inflammation, and thrombosis have been identified as mechanisms involved in the formation of atherosclerotic plaques [2]. The pathogenesis of atherosclerosis seems to be causally linked to an imbalance between the production of reactive oxygen species (ROS) and the available antioxidant defense systems [3,4]. The resulting oxidative stress leads to cell damage by the direct oxidation of cellular proteins, lipids, and DNA or via cell death signaling pathways responsible for accelerating atherogenesis [4]. One of several promising targets against the progression of inflammatory vascular diseases including atherosclerosis represents the inhibition of endothelial cell apoptosis [5,6,7].

An important antioxidant, anti-inflammatory, and cytoprotective enzyme is heme oxygenase-1 (HO-1), which catalyzes the degradation of heme to form biliverdin, iron ions, and carbon monoxide (CO) and is induced by oxidative stress [8]. Among other properties, HO-1 inhibits the formation of ROS and tumor necrosis factor-mediated apoptosis of endothelial cells [9,10] and exerts a protective effect on endothelial cells exposed to hyperglycemic and oxidative/nitrosative stress conditions [11,12]. In addition, several studies have pointed to a critical role of HO-1 in the regulation of autophagy, which has been associated with survival-enhancing effects in various cell types, including endothelial cells [13,14,15,16]. Finally, HO-1 showed in vivo positive effects in animal models of atherosclerosis and restenosis [17]. On the other hand, HO-1 gene polymorphisms, more precisely a larger number of guanosine thymidine dinucleotide repeats in the HO-1 gene promoter, have been associated with a higher risk of chronic renal disease, cardiovascular events, and mortality in patients with coronary heart disease [18]. However, some reports also suggest that excessive and prolonged HO-1 expression leads to negative effects on cell function and survival [19,20]. In this context, the cytotoxic component is attributed to the release of molecular iron or high concentrations of CO, an inhibitor of the respiratory chain [21,22,23,24].

For the treatment of diseases associated with atherosclerosis, interest in the non-psychotropic cannabis-based active substance cannabidiol (CBD) has increased significantly in recent years. Indeed, CBD has been shown to suppress a high glucose-induced inflammatory response and barrier disruption of endothelial cells [25] and to attenuate myocardial dysfunction, cardiac fibrosis, oxidative/nitrative stress, inflammation, cell death, and interrelated signaling pathways in a mouse model of type I diabetic cardiomyopathy [26]. Although the hypothesis of an initiation of antioxidative signaling pathways has been repeatedly raised for CBD [25,26,27], the influence of the phytocannabinoid on endothelial survival has hardly been investigated.

In the search for a possible target for CBD in the prevention of endothelial cell death, we have focused on the enzyme HO-1. Accordingly, the present study investigated the effect of CBD on the expression of HO-1 in human umbilical vein endothelial cells (HUVEC) and the associated changes in autophagy and apoptosis. For the first time, we provide evidence for the ROS-dependent induction of HO-1 expression in endothelial cells as a mechanism by which CBD mediates protective autophagy. However, at higher CBD concentrations (>6 µM) and thereby resulting in very high HO-1 levels, this protective effect is no longer able to prevent the induction of cell death inducing apoptosis.

## 2. Materials and Methods

### 2.1. Materials

CBD was supplied by Biotrend (Cologne, Germany). AM-251 and AM-630 were bought from Biomol GmbH (Hamburg, Germany). Capsazepine and N-acetyl-L-cysteine were from Sigma-Aldrich (Taufkirchen, Germany). Bafilomycin A_1_ was obtained from InvivoGen (Toulouse, France). HUVEC, endothelial cell growth medium (ECGM), and supplements were obtained from PromoCell GmbH (Heidelberg, Germany). Nrf2 siRNA (sc-37030), Nrf2 (C-20) antibody (sc-722), and copper protoporphyrin IX (CuPPIX) were purchased from Santa Cruz Biotechnology, Inc. (Heidelberg, Germany). HO-1 antibody (ADI-SPA-895) and tin protoporphyrin IX (SnPPIX) were obtained from Enzo Life Sciences GmbH (Lörrach, Germany). Cleaved caspase-3 (Asp175) (5A1E) antibody (#9664), LC3A/B antibody (#4108), and secondary antibodies (anti-rabbit antibody, #7074; anti-mouse antibody, #7076) were purchased from Cell Signaling Technology Europe (Frankfurt/Main, Germany). β-Actin antibody (clone AC-74, #A5316) was obtained from Sigma-Aldrich (Taufkirchen, Germany). Negative control siRNA (cat. no. 1022076) was from Qiagen (Hilden, Germany). Transfection reagent Lipofectamine^TM^ RNAiMAX and transfection medium Opti-MEM^®^ I Reduced Serum Medium were obtained from Thermo Fisher Scientific Inc. (Schwerte, Germany).

### 2.2. Cell Culture

HUVEC were maintained in endothelial cell growth medium (ECGM) supplemented with 0.4% endothelial cell growth supplement (ECGS), 2% fetal calf serum (FCS), 0.1 ng/mL epidermal growth factor (EGF), 1 ng/mL basic fibroblast growth factor (bFGF), 90 μg/mL heparin, and 1 μg/mL hydrocortisone (all from Promocell). The cells were grown in a humidified incubator at 37 °C and 5% CO_2_. Experiments were performed using HUVEC at passages 2 to 6. All incubations were performed in complete medium. NAC was diluted in phosphate-buffered saline. All other test substances were dissolved in ethanol, DMSO, or NaOH, with the corresponding solvents showing final concentrations in the incubates of maximal 0.033% (*v*/*v*) ethanol (for 10 µM CBD), 0.025% (*v*/*v*) DMSO (for CuPPIX), 0.01% (*v*/*v*) DMSO (for AM-251, AM-630, capsazepine), 0.05% (*v*/*v*) DMSO (for 50 nM bafilomycin A_1_), or 0.001 M NaOH (for SnPPIX). The respective vehicle control incubate contained the corresponding concentration of ethanol, DMSO, or NaOH of the test substance incubates.

### 2.3. Cell Viability Analysis

The viability of the cells was determined using the colorimetric WST-1 test (Roche Diagnostics, Mannheim, Germany), in which the water-soluble tetrazolium salt WST-1 is bioreduced by NAD(P)H to a formazan dye. Accordingly, the amount of formazan dye formed correlates directly with the metabolic activity of the cells. Cells were seeded into 24-well plates at 1 × 10^5^ cells per well, with the exception of the experiment on the concentration-dependent influence of CBD (0.1 to 10 µM) on viability after 48 h, for which cells were seeded into 96-well plates with 5 × 10^3^ cells per well. After 24 h, medium was changed, and cells were treated with the respective test substances for the indicated times. After the respective incubation time, cell viability was measured. In co-incubation experiments using SnPPIX, the medium was refreshed prior to the addition of WST-1 reagent to avoid influences in absorbance measurement due to the coloring of SnPPIX.

### 2.4. Quantitative RT-PCR Analysis

HUVEC seeded into 24-well plates with a density of 1 × 10^5^ cells per well were grown to confluence. The cells were incubated with the respective test substances or their vehicles for the specified times. Then, cell culture media were removed, and the cells were lysed for RNA isolation. The total RNA was isolated with the RNeasy total RNA Kit (Qiagen, Hilden, Germany). According to the manufacturer’s instructions, β-actin (internal standard) and HO-1 mRNA levels were determined with the TaqMan^®^ RNA-to-CT™ 1-Step Kit (Applied Biosystems, Darmstadt, Germany) by means of quantitative real-time RT-PCR. HO-1 mRNA levels were normalized to β-actin, and samples were compared to appropriate vehicle controls. Primers and probes for human β-actin and HO-1 were TaqMan^®^ Gene Expression Assay products (Applied Biosystems, Darmstadt, Germany).

### 2.5. Western Blot Analysis

For the analysis of HO-1, Nrf2, caspase-3, LC3A/B-I/II, and β-actin at the protein level, HUVEC were seeded in 24-well or 6-well plates with a density of 1 × 10^5^ or 4 × 10^5^ cells per well. After 24 h, the medium was changed. After incubation with the test substances or their vehicles for the specified times, cell culture media (non-adherent cells) and trypsinated (adherent) cells were collected per well of a 6-well plate or of 4 pooled wells with the same treatment of a 24-well plate and centrifuged at 500× *g*. Each cell pellet was lysed in 50 µL sample buffer, boiled at 95 °C for 5 min, homogenized by sonication, and centrifuged at 10,000× *g* for 5 min. Supernatants were used for Western blot analysis. Total protein in supernatants was measured using a Pierce™ bicinchoninic acid (BCA) protein assay kit (Thermo Fisher Scientific Inc., Schwerte, Germany) according to the manufacturer’s protocol.

Then, equal amounts of denatured proteins were separated on a 12% sodium dodecyl sulfate–polyacrylamide gel. After transfer to nitrocellulose and blocking of the membranes with 5% milk powder, the blots were probed with specific primary antibodies. To detect the corresponding proteins, the membranes were probed with horseradish peroxidase-conjugated rabbit or mouse secondary antibodies. Visualization of antibody binding was performed using a chemiluminiferous solution (100 mM Tris-HCl pH 8.5, 1.25 mM luminol, 200 µM p-coumaric acid, 0.09% (*v*/*v*) hydrogen peroxide, 0.0072% (*v*/*v*) DMSO). Densitometric analysis of band intensities was conducted by optical scanning and quantification with Quantity One 1-D Analysis Software (Biorad, Munich, Germany). After the analysis was completed, membranes were stripped and reprobed. Protein expression was normalized to β-actin and compared to the corresponding vehicle controls.

### 2.6. siRNA Transfection

Reverse transfection of siRNA targeting Nrf2 mRNA was performed according to the manufacturer’s instructions (Thermo Fisher Scientific Inc., Schwerte, Germany). In brief, 3.5 × 10^4^ cells in 0.5 mL basal endothelial growth medium per well were added to 0.1 mL per well of transfection medium Opti-MEM^®^ I Reduced Serum Medium containing a mixture of siRNA (final concentration in incubates: 20 nM) and siRNA transfection reagent Lipofectamine^TM^ RNAiMAX (1 µL), and mixed and incubated for 24 h in a 24-well plate. Thereafter, the medium was changed, and cells were incubated with vehicle or CBD for another 24 h. Cells were transfected with non-silencing control siRNA (20 nM) in parallel to demonstrate specific gene silencing. Subsequently, the cell viability tests and Western blot analyses were performed as described above.

### 2.7. Statistics

Comparisons between groups were performed with Student´s two-tailed *t* test or with one-way ANOVA with Bonferroni´s (selected comparisons) or Dunnett´s post hoc test using GraphPad Prism 5.00 (GraphPad Software, San Diego, CA, USA). In the case of Bonferroni’s post hoc test, the determination of statistical significance was limited to the groups of interest for reasons of clarity of presentation. Results were considered to be statistically significant at values of *p* < 0.05 and were designated in the figures accordingly.

## 3. Results

### 3.1. CBD Causes a Concentration- and Time-Dependent Induction of HO-1 Expression in HUVEC

**Figure 1:**
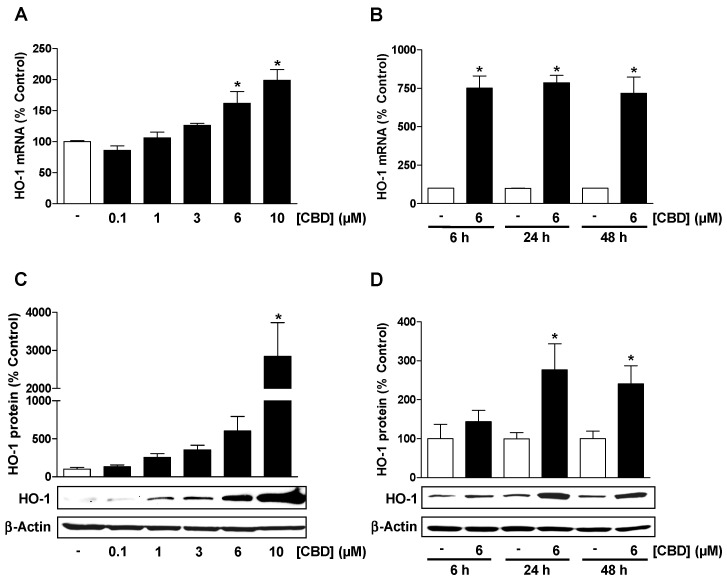
Cannabidiol (CBD) causes a concentration- and time-dependent induction of heme oxygenase-1 (HO-1) expression in human umbilical vein endothelial cells (HUVEC). Concentration-dependent effect of CBD on HO-1 mRNA (**A**) and HO-1 protein (**C**) expression following incubation with CBD or vehicle for 24 h. Time-dependent effect of CBD on HO-1 mRNA (**B**) and HO-1 protein (**D**) expression following incubation with CBD or vehicle for the times indicated. Expression values were normalized to β-actin. Percent control represents comparison with vehicle-treated cells (100%) in the absence of test substance. Values are means ± SEM of n = 4 (**A**), n = 3 (**B**), n = 6 (**C**), or n = 5 (**D**) experiments. The values for blots were determined by densitometric analysis. Representative blots are shown. * *p* < 0.05 vs. corresponding time-matched vehicle control; one-way ANOVA with Dunnett´s post hoc test (**A**,**C**) or Student´s two-tailed *t* test (**B**,**D**).

To determine whether CBD increases HO-1 expression in HUVEC, cells were treated with the substance for 6 to 48 h. As shown in Figure 1A,B, incubation of cells with CBD at concentrations up to 10 µM was associated with a concentration-dependent increase in HO-1 mRNA and a constantly high mRNA increase in the range of 6 to 48 h. A concentration-dependent increase was also registered for the HO-1 protein (Figure 1C), with CBD causing a corresponding maximum after 24 h (Figure 1D).

### 3.2. Reactive Oxygen Species but not Cannabinoid-Activated Receptors Mediate CBD-Induced HO-1 Expression in HUVEC

**Figure 2:**
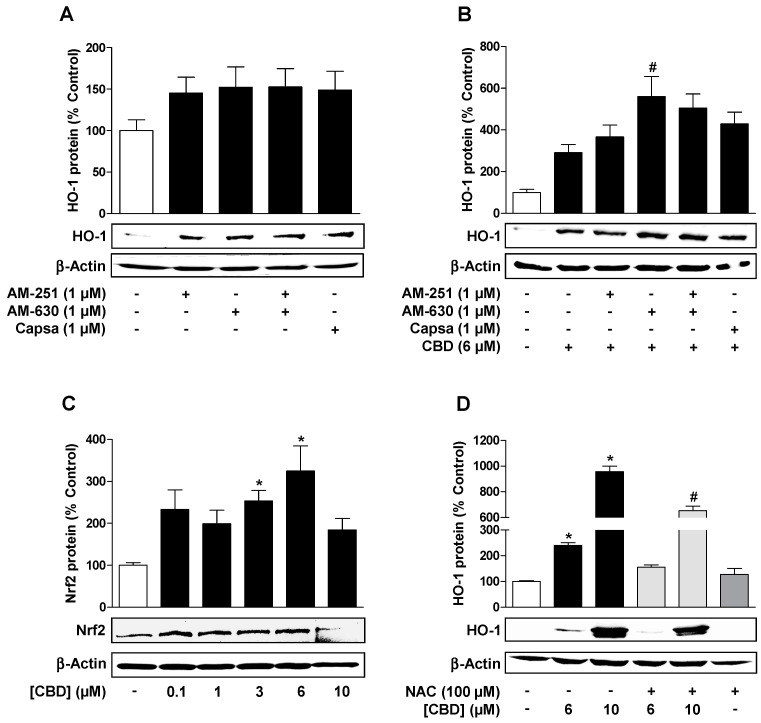
Reactive oxygen species (ROS) but not cannabinoid-activated receptors mediate CBD-induced HO-1 expression in HUVEC. Effect of AM-251 (CB_1_ antagonist), AM-630 (CB_2_ antagonist), and capsazepine (Capsa; transient receptor potential vanilloid 1 (TRPV1) antagonist) on HO-1 protein expression alone (**A**) or in combination with CBD (**B**). Cells were pre-incubated with the respective receptor antagonist (all tested at a final concentration of 1 µM) for 30 min and then further co-incubated with CBD (6 µM) for another 24 h. (**C**) Concentration-dependent effect of CBD on Nrf2 expression in HUVEC following incubation with CBD or vehicle for 24 h. (**D**) Effect of the antioxidant and ROS scavenger N-acetyl-L-cysteine (NAC) on CBD-induced HO-1 expression. Cells were pre-incubated with 100 µM NAC for 30 min and then further co-incubated with CBD for another 24 h. Expression values were normalized to β-actin. Percent control represents comparison with vehicle-treated cells (100%) in the absence of test substance. Values are means ± SEM obtained from densitometric analysis of n = 7 (**A**), n = 12 (**B**), n = 4 (**C**), and n = 3 (**D**) experiments. The values for blots were determined by densitometric analysis. Representative blots are shown. * *p* < 0.05 vs. corresponding vehicle control, *# p* < 0.05 vs. corresponding CBD-treated group; one-way ANOVA with Bonferroni´s (**B**,**D**) or Dunnett´s (**C**) post hoc test.

After demonstrating a concentration-dependent increase in HO-1 expression by CBD (Figure 1), a possible role of CB receptors and the transient receptor potential vanilloid 1 (TRPV1) in HO-1 induction by 6 µM CBD was next investigated. For this purpose, cells were pre-incubated with the CB_1_ receptor antagonist AM-251, the CB_2_ receptor antagonist AM-630, or the TRPV1 antagonist capsazepine. All antagonists were used at a concentration of 1 μM, which is in the range of concentrations that inhibit CB_1_-, CB_2_-, and TRPV1-dependent events [28,29,30,31] and had no significant influence on HO-1 expression in our experiments (Figure 2A). However, none of the three substances led to an inhibition of CBD-induced HO-1 induction (Figure 2B), suggesting receptor-independent events as the underlying mechanism. In accordance with these data, the receptor antagonists were also unable to prevent HO-1 induction by 10 µM CBD (data not shown).

It is known that the activation of nuclear factor erythroid 2-related factor 2 (Nrf2) is involved in the increased expression of HO-1 [32,33]. To prove the influence of CBD on the transcription factor Nrf2, its protein content was analyzed after 24 h treatment with concentrations up to 10 µM CBD. Here, CBD induced an upregulation of the Nrf2 protein when using concentrations up to 6 µM, while the Nrf2 levels induced by 10 µM CBD decreased compared to the Nrf2 expression levels induced by 6 µM CBD (Figure 2C). A participation of ROS in CBD-induced HO-1 expression was analyzed with the antioxidant and ROS scavenger N-acetyl-L-cysteine (NAC). The cells were pre-incubated with NAC for 30 min and then further co-incubated with the indicated CBD concentration (Figure 2D). NAC significantly reduced the HO-1 induction of 10 µM CBD and led to an approximate 60% reduction in HO-1 protein levels induced by 6 µM CBD, indicating a participation of ROS in HO-1 induction (Figure 2D).

### 3.3. CBD Induces a Concentration-Dependent Increase in Cellular Autophagy, but Regulates Metabolic Activity and Apoptosis Differently Depending on the Concentration

**Figure 3:**
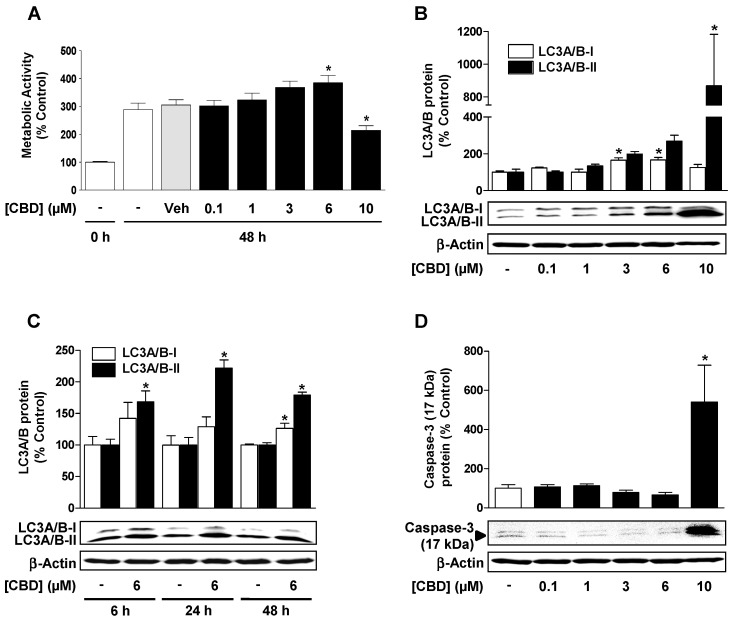
CBD induces a concentration-dependent increase in cellular autophagy, but it regulates metabolic activity and apoptosis differently depending on the concentration. (**A**) Concentration-dependent effect of CBD on viability of HUVEC. Concentration- (**B**) and time-dependent (**C**) effect of CBD on light chain 3 A (LC3A)/B-I/II expression. (**D**) Concentration-dependent effect of CBD on caspase-3 cleavage. Cells were incubated with CBD for 48 h (**A**), 24 h (**B**,**D**) or for the times indicated (**C**). Percent control represents comparison with vehicle-treated cells (100%) in the absence of test substance, in (**A**) with non-treated cells at 0 h (100%). Expression values were normalized to β-actin. Values are means ± SEM of n = 20–21 (**A**), n = 6 (**B**), n = 3 (**C**), or n = 10 (**D**) experiments. The values for blots were determined by densitometric analysis. Representative blots are shown. * *p* < 0.05 vs. corresponding vehicle control, in (**A**) vs. vehicle (Veh) at 48 h; one-way ANOVA with Dunnett´s post hoc test (**A**,**B**,**D**) or Student´s two-tailed *t* test (**C**).

The functional significance of the induction of HO-1 by CBD was consequently investigated. To demonstrate the influence of CBD on the metabolic activity (viability) of HUVEC, the cells were incubated with the substance in concentrations of 0.1 to 10 µM for 48 h and then subjected to a WST-1 assay. As shown in Figure 3A, the metabolic activity of HUVEC was increased after 48 h incubation with CBD at concentrations of 1 to 6 µM, whereby the effect of 6 µM CBD was significant. In contrast, 48-h treatment of HUVEC with 10 µM CBD resulted in a significant decrease in metabolic activity compared to the corresponding vehicle control (Figure 3A).

In order to investigate autophagy as a potential CBD-induced protective mechanism, HUVEC were incubated with CBD in the same concentration range (0.1–10 µM) that was used for HO-1 expression analysis. For the initiation of autophagy, the conjugation of the microtubule-associated protein 1 light chain 3-I (LC3-I) with phosphatidylethanolamine (PE) is required. The LC3-II generated in this way is responsible for the maturation of the autophagosomes [34,35]. Accordingly, LC3-I and LC3-II levels were determined by Western blot. As shown in Figure 3B, CBD led to a concentration-dependent induction of autophagy, which resulted in a significant increase in LC3A/B-I and LC3A/B-II protein expression. The decisive LC3A/B-II protein expression by 6 µM CBD was time-dependent and showed maximum stimulation values after an incubation period of 24 h (Figure 3C). The activation of autophagy was evaluated by assessing the expression of the PE-conjugated LC3A/B-II protein normalized to β-actin instead of the protein ratio between LC3-I and LC3-II, since different affinities of antibodies against LC3-I and LC3-II and different expression levels of these proteins depending on the cell line and tissue have been reported in the literature [35,36]. Although the incubation of HUVEC with 10 µM CBD induced excessive LC3A/B-II protein expression (Figure 3B), a significant increase in caspase-3 apoptosis marker (Figure 3D) was detected at the same time, which correlated with a decrease in metabolic activity (Figure 3A).

### 3.4. ROS Mediate the CBD (10 µM) Induced Reduction of Metabolic Activity as well as the Increase of Autophagy and Induction of Apoptosis in HUVEC

**Figure 4:**
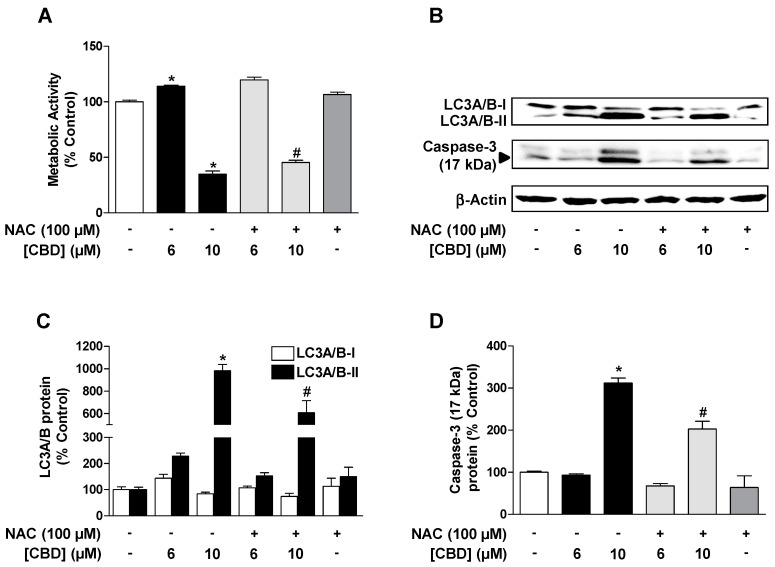
ROS mediate the CBD (10 µM)-induced reduction of metabolic activity as well as the increase of autophagy and induction of apoptosis in HUVEC. Effect of N-acetyl-L-cysteine (NAC) on CBD effects on viability (**A**), LC3A/B-I/II expression (**B**,**C**), and caspase-3 cleavage (**B**,**D**) in HUVEC. Cells were pre-incubated with 100 µM NAC for 30 min and then further co-incubated with CBD for another 24 h. Expression values were normalized to β-actin. Percent control represents comparison with vehicle-treated cells (100%) in the absence of test substance. Values are means ± SEM of n = 4 (**A**) or n = 3 (**C**,**D**). The values for blots were determined by densitometric analysis. Representative blots are shown in (**B**). * *p* < 0.05 vs. corresponding vehicle control, *# p* < 0.05 vs. corresponding CBD-treated group; one-way ANOVA with Bonferroni´s post hoc test.

Since CBD-induced HO-1 expression is ROS-dependent as described above, the influence of ROS on LC3A/B-I/II and cleaved caspase-3 as well as on metabolic activity was next investigated with the ROS scavenger NAC. For this purpose, the cells were pre-incubated with NAC for 30 min and then further co-incubated with CBD. As a result of these studies, NAC was shown to significantly reduce the 10 µM CBD-induced decrease in metabolic activity (Figure 4A) as well as LC3A/B-I/II (Figure 4B,C) and caspase-3 expression (Figure 4B,D). Consequently, 10 µM CBD induces ROS-dependent autophagy and apoptosis induction, leading to a loss of HUVEC viability at this concentration. With regard to the non-proapoptotic 6 µM concentration of CBD (Figure 4D), NAC caused an approximate 60% inhibition of LC3A/B-II expression (Figure 3C); however, this was not significant and showed no inhibitory effect on CBD-induced metabolic activity (Figure 4A).

### 3.5. Inhibition of CBD-Induced Autophagy Leads to Increased Apoptosis and Loss of Viability of HUVEC

**Figure 5:**
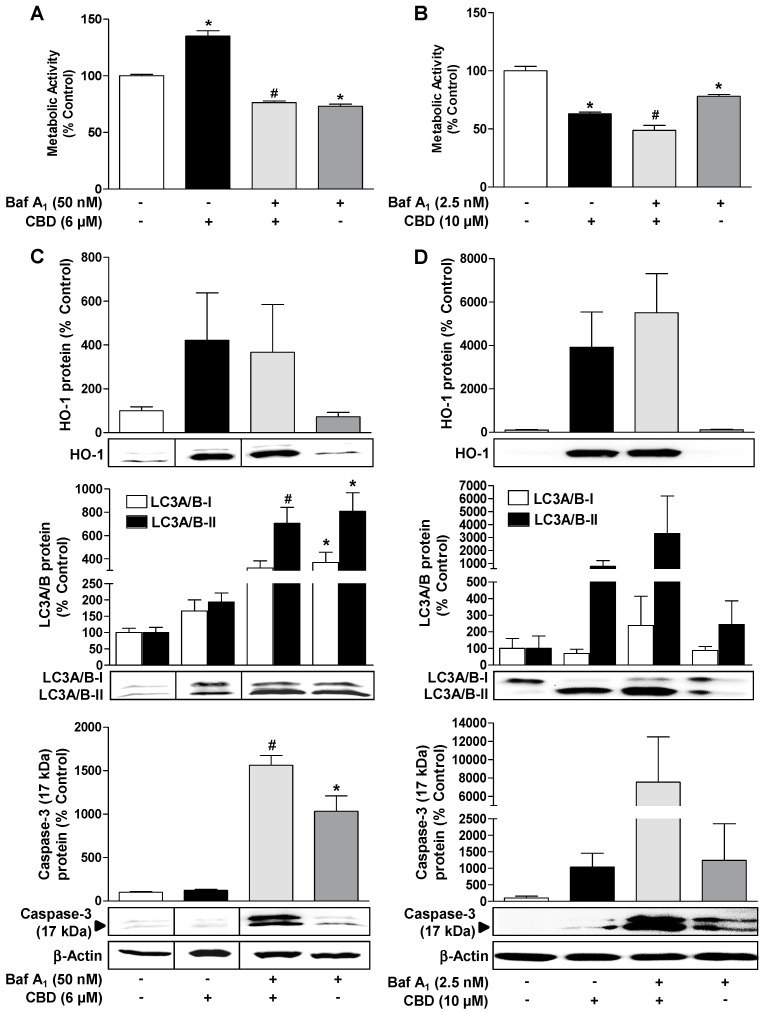
Inhibition of CBD-induced autophagy leads to increased apoptosis and loss of viability of HUVEC. Influence of bafilomycin A_1_, an inhibitor of autophagic flux, on CBD effects on viability (**A**,**B**), HO-1 expression (**C**,**D**, upper blots), LC3A/B-I/II expression (**C**,**D**, middle blots), and caspase-3 cleavage (**C**,**D**, lower blots) in HUVEC. The cells were pre-incubated with 50 nM (**A**,**C**) or 2.5 nM (**B**,**D**) bafilomycin A_1_ (Baf A_1_) for 30 min and then further co-incubated with 6 µM (**A**,**C**) or 10 µM CBD (**B**,**D**) for another 24 h. Expression values were normalized to β-actin. The vertical black lines inside the boxes of some blots (**C**) indicate that the lanes in between have been removed, so here too, signals from protein samples loaded onto the same gel were compared. The actin blots shown in **C** and **D** were used as controls for the 3 blots shown above, since the proteins analyzed in these Western blottings were resolved on the same gel. Percent control represents comparison with vehicle-treated cells (100%). Values are means ± SEM of n = 4 (**A**,**B**), n = 6 (**C**, upper blots), n = 7 (**C**, middle and lower blots) or n = 3 (**D**) experiments. The values for blots were determined by densitometric analysis. Representative blots are shown. * *p* < 0.05 vs. corresponding vehicle control, *# p* < 0.05 vs. corresponding CBD-treated group; one-way ANOVA with Bonferroni´s post hoc test.

To investigate the role of autophagy in preventing apoptosis of HUVEC under basal and CBD-modulated conditions, further experiments with the autophagy inhibitor bafilomycin A_1_ were performed. Bafilomycin A_1_ led to a profound caspase-3 cleavage in the presence of 6 µM and 10 µM CBD (Figure 5C,D, lower blots), indicating a protective effect of autophagy on the proapoptotic potential of CBD.

In the case of the experiments with 10 µM of CBD, bafilomycin A_1_ in a relatively low concentration of 2.5 nM was used to prevent secondary necrosis as a consequence of a very strong induction of apoptosis by 10 µM CBD in the presence of the autophagy inhibitor.

In parallel experiments, it was shown that bafilomycin A_1_ led to a significant inhibition of cell viability both under basal conditions and when administered concomitantly with CBD, showing an important role of autophagy in maintaining homeostasis in HUVEC (Figure 5A,B). The inhibition of autophagous flow by bafilomycin A_1_ had no significant effect on HO-1 expression (Figure 5C,D, upper blots). According to bafilomycin A_1_-induced inhibition of the fusion of autophagosomes with lysosomes, which normally stimulates the degradation of LC3-II, the inhibitor led to an increase in the LC3A/B-II protein when administered alone or in combination with CBD (Figure 5C,D, middle blots).

### 3.6. Inhibition of HO-1 Activity by SnPPIX Reduces CBD-Induced Autophagy and Attenuates the Loss of Viability Due to 10 µM CBD

**Figure 6:**
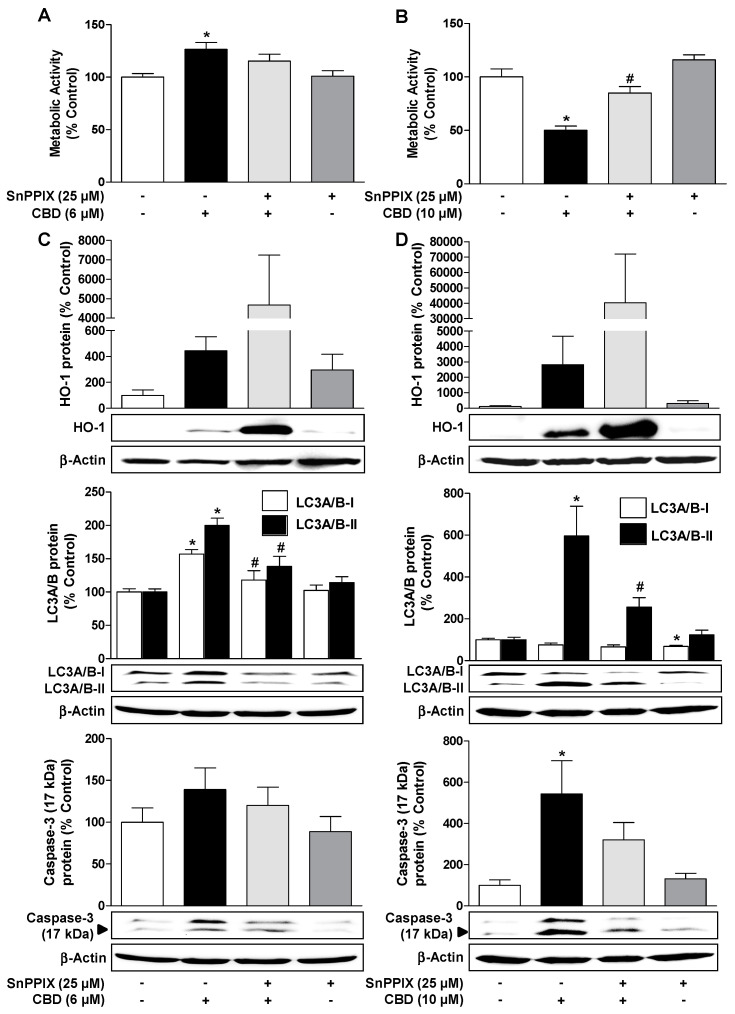
Inhibition of HO-1 activity by tin protoporphyrin IX (SnPPIX) reduces CBD-induced autophagy and attenuates the loss of viability due to 10 µM CBD. Effect of the HO-1 inhibitor SnPPIX on CBD effects on viability (**A**,**B**), HO-1 expression (**C**,**D**, upper blots), LC3A/B-I/II expression (**C**,**D**, middle blots) and caspase-3 cleavage (**C**,**D**, lower blots) in HUVEC. The cells were pre-incubated with 25 µM SnPPIX for 30 min and then further co-incubated with 6 µM (**A**,**C**) or 10 µM CBD (**B**,**D**) for another 24 h. Expression values were normalized to β-actin. The same actin blot was used as a control for LC3A/B and caspase-3 in (**C**) and (**D**), since these proteins were resolved on the same gel. Percent control represents comparison with vehicle-treated cells (100%) in the absence of test substance. Values are means ± SEM of n = 6 (**A**), n = 4 (**B**,**C**, upper and middle blots, **D**, middle and lower blots), n = 9–10 (**C**, lower blots), or n = 3 (**D**, upper blots) experiments. The values for blots were determined by densitometric analysis. Representative blots are shown. * *p* < 0.05, vs. corresponding vehicle control, # *p* < 0.05 vs. CBD-treated group; one-way ANOVA with Bonferroni´s post hoc test.

To clarify a possible correlation between HO-1 induction by CBD and the associated autophagic effect, it was experimentally tested whether the HO inhibitor SnPPIX can reverse the proautophagic effect of CBD. As shown in Figure 6C,D (middle blots), SnPPIX significantly inhibited the LC3A/B-II protein expression increased by both 6 µM and 10 µM CBD. Furthermore, SnPPIX treatment also reduced caspase-3 activation (Figure 6C,D, lower blots). SnPPIX further led to a significant inhibition of the viability-reducing effect of 10 µM CBD (Figure 6B) and reduced the viability-increasing effect of 6 µM CBD by approximately 43% (Figure 6A). The above effects of the HO-1 inhibitor SnPPIX were observed despite increased HO-1 protein expression by SnPPIX alone or when incubated together with CBD (Figure 6C,D, upper blots).

To confirm the specific inhibition of HO-1 activity by SnPPIX, parallel experiments were performed with CuPPIX, a non-HO-1-inhibiting structural analogue of SnPPIX. CuPPIX alone also induced the HO-1 protein, and even co-treatment with CBD led to a further upregulation of the HO-1 protein (Table 1). However, in contrast to SnPPIX, CuPPIX led to an induction of LC3A/B-I and LC3A/B-II protein expression and caspase-3 cleavage when incubated alone, or to an additive induction of LC3A/B-I and LC3A/B-II protein expression and caspase-3 cleavage when incubated with CBD (Table 1). These results confirm the specific inhibition of HO-1 activity by SnPPIX.

### 3.7. Inhibition of HO-1 Expression by Nrf2 siRNA Reduces CBD-Induced Autophagy and Attenuates the Loss of Viability by 10 µM CBD

**Figure 7:**
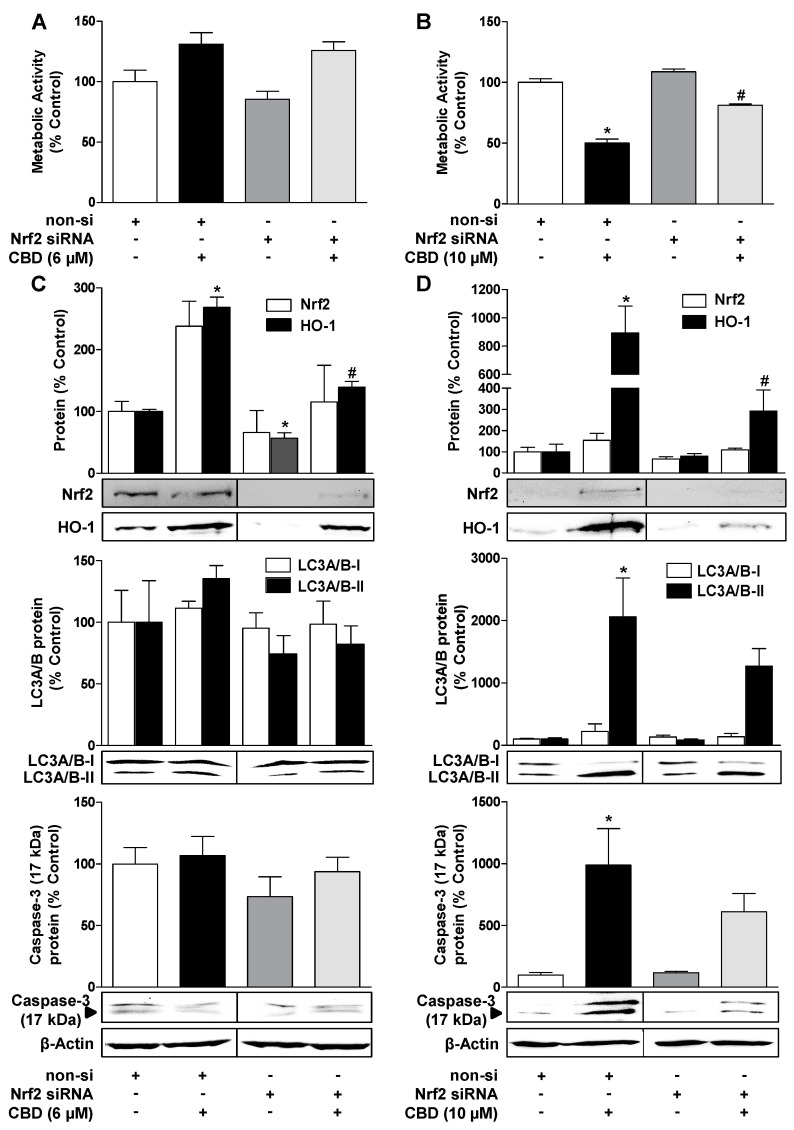
Inhibition of HO-1 expression by nuclear factor erythroid 2-related factor 2 (Nrf2) siRNA reduces CBD-induced autophagy and attenuates the loss of viability by 10 µM CBD. Effect of Nrf2 siRNA compared to non-silencing (non-si) control siRNA on CBD effects on viability (**A**,**B**), Nrf2 and HO-1 expression (**C**,**D**, upper blots), LC3A/B-I/II expression (**C**,**D**, middle blots), and caspase-3 cleavage (**C**,**D**, lower blots) in HUVEC. The cells were transfected with selective Nrf2 siRNA or non-silencing siRNA. Subsequently, vehicle, 6 µM CBD (**A**,**C**), or 10 µM CBD (**B**,**D**) was added, and the incubation was continued for another 24 h. Expression values were normalized to β-actin. The vertical black lines inside the boxes of the blots indicate that the blots were rearranged at these points, so here too, signals from protein samples loaded onto the same gel were compared. The actin blots shown in (**C**) and (**D**) were used as controls for the 4 blots shown above, since the proteins analyzed in these Western blottings were resolved on the same gel. Values are means ± SEM of n = 4 (**A**,**B**), n = 3 (**C**, upper blots, Nrf2), n = 5 (**C**, upper blots, HO-1), n = 4 (**C**, middle and lower blots, **D**, upper blots, Nrf2) or n = 7 (**D**, upper blots, HO-1, middle and lower blots) experiments. The values for blots were determined by densitometric analysis. Representative blots are shown. * *p* < 0.05 vs. corresponding vehicle control, # *p* < 0.05 non-si siRNA group vs. corresponding Nrf2 siRNA group; one-way ANOVA with Bonferroni´s post hoc test.

To further confirm the functional role of HO-1 in CBD-mediated autophagy, HO-1 should be downregulated with selective Nrf2 siRNA in subsequent experiments. Relative to cells transfected with control siRNA, Nrf2 siRNA inhibited the upregulation of Nrf2 and significantly reduced HO-1 expression induced by 6 µM and 10 µM CBD (Figure 7C,D, upper blots).

At the functional level, the significantly decreased HO-1 expression caused by Nrf2 siRNA was associated with a reduction of the proautophagic effects of 6 µM and 10 µM CBD and the proapoptotic action of 10 µM CBD compared to CBD-treated cells transfected with non-silencing siRNA (Figure 7C,D, middle and lower blots). In accordance with the data obtained with SnPPIX, Nrf2 siRNA led to a significant inhibition of the 10 µM CBD mediated decrease in the vitality of HUVEC transfected with non-silencing siRNA (Figure 7B), but it caused only about 17% inhibition of the 6 µM CBD induced increase in metabolic activity (Figure 7A).

## 4. Discussion

**Figure 8:**
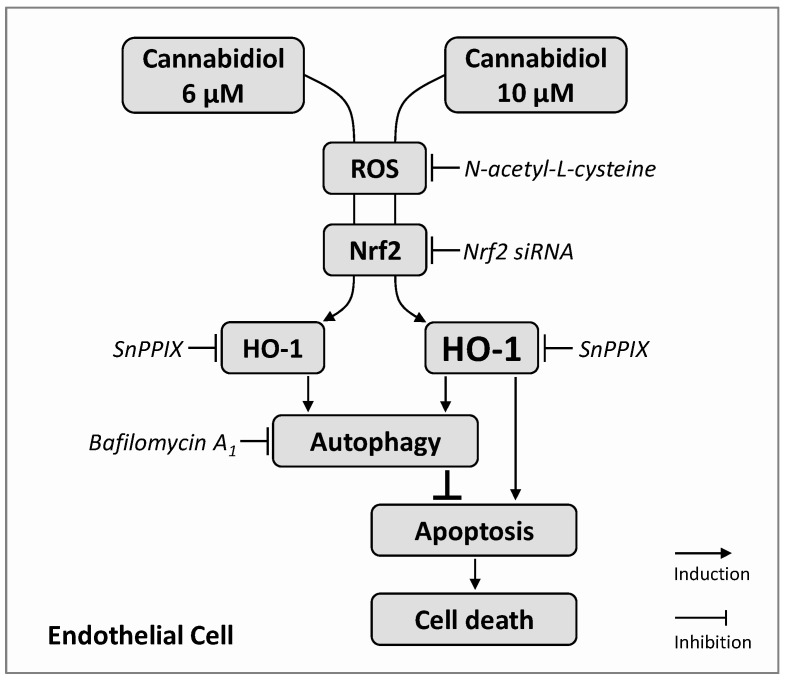
Proposed mechanism and functional consequence underlying cannabidiol (CBD)-induced HO-1 expression in HUVEC. CBD leads to a concentration-dependent increase in HO-1 expression, which is mediated by reactive oxygen species (ROS) and the transcription factor Nrf2, but not by cannabinoid-activated membrane receptors (CB_1_, CB_2_, TRPV1). HO-1 subsequently initiates proautophagic processes that confer anti-apoptotic and life-promoting effects. However, above a critical concentration, as in the case of 10 µM CBD, HO-1 leads to the induction of cellular apoptosis. Furthermore, the inhibition of HO-1-dependent CBD-induced autophagy leads to apoptosis induction by a per se viability-promoting CBD concentration (6 µM) or to superinduction of apoptosis by a per se cytotoxic CBD concentration (10 µM). The sequence shown with inductive and inhibitory arrows connecting the boxes corresponds to the effect of the CBD in the absence of inhibitors of the individual members of the signal transduction. The corresponding inhibitors are shown in italics, whereby their target, but not their consecutive effect, is shown.

The present study demonstrates the Nrf2- and ROS-dependent induction of HO-1 expression in endothelial cells as a mechanism by which the non-psychoactive cannabinoid CBD mediates concentration-dependent autophagy and, at higher concentrations, apoptosis independent of the activation of cannabinoid receptors and TRPV1 (Figure 8).

A large body of evidence supports this finding. First, CBD at concentrations between 1 and 10 µM caused a concentration-dependent upregulation of HO-1 mRNA, HO-1 protein, and Nrf2 in HUVEC. Secondly, a proautophagic effect of CBD was observed at final concentrations of 3 to 10 µM, whereby the protective role was underlined by experiments with the autophagy inhibitor bafilomycin A_1_, which led to apoptosis induction or superinduction in cells treated with 6 µM or 10 µM CBD, respectively. Thirdly, the inhibition of HO-1 by SnPPIX as well as the siRNA-mediated knockdown of HO-1 by Nrf2 siRNA led to an inhibition of the proautophagic effect of 6 and 10 µM CBD and the apoptosis-inducing and viability-reducing effect of 10 µM CBD. Fourth, the involvement of cannabinoid-activated receptors (CB_1_, CB_2_, TRPV1) in CBD-induced HO-1 expression was excluded by the use of selective receptor antagonists.

In accordance with our results, several studies have shown that the activation of Nrf2 is associated with an accumulation of Nrf2 in the entire cell lysate [32,33,37,38] and that the release of Nrf2 from Keap1 repression by e.g., ROS leads to the stabilization and subsequent translocation of Nrf2 into the cell nucleus and to the activation of HO-1 transcription [39]. Regarding the demonstrated HO-1 induction by CBD, further investigations of our group have shown that this stimulation is not restricted to endothelial cells but also occurs in vascular smooth muscle cells [40] or in adipose-derived mesenchymal stem cells (unpublished results), whereby in the mentioned cell types, HO-1 induction was also registered in the presence of Δ^9^-tetrahydrocannabinol (THC), which is another phytocannabinoid. Furthermore, the induction of HO-1 expression by CBD in microglial cells [41,42] and keratinocytes [43] as well as by CB_2_ agonists in Kupffer cells [44] and in myocardium [45,46] was reported, whereas the incubation of glioma cells with the CB_2_ agonist JWH-133 led to a reduced HO-1 gene expression [47]. In accordance with our data, a receptor-independent but ROS-dependent upregulation of the HO-1 signaling pathway was finally also observed in breast cancer cells when fatty acid amide hydrolase, an important endocannabinoid-degrading enzyme, was inhibited or when the cells were exposed to the endocannabinoid anandamide [48].

In agreement with our finding demonstrating CBD-induced and ROS/HO-1-mediated protective autophagy in endothelial cells, other studies have also shown that intracellular redox status exerts significant effects on the autophagy process of endothelial cells. Thus, an inhibitory effect of antioxidants on the autophagy response activated by various stimuli has been reported repeatedly [49,50]. Furthermore, there is evidence of complex interactions between autophagy and other stress processes, such as HO-1 induction [51,52]. For this reason, the HO inhibitor SnPPIX was used to investigate a possible relationship between the induction of HO-1 by CBD and its proautophagic effect. The interpretation of these results was complicated by the fact that SnPPIX itself caused an upregulation of HO-1 expression in HUVEC, which led to an overadditive increase of HO-1 expression in the presence of CBD. On the other hand, the induction of HO-1 transcription by SnPPIX, which is well described in the literature, is relativized by the fact that under these circumstances, the HO inhibitor still mediates the sufficient blocking of the activity of the preformed and de novo synthesized HO-1 enzyme [53,54]. Irrespective of this, we were able to exclude possible off-target effects of SnPPIX by testing a negative control, namely the non-HO-1-inhibiting structural analogue CuPPIX. Furthermore, a causal relationship between HO-1 induction and autophagy could be confirmed by using Nrf2 siRNA, which led to a downregulation of HO-1 expression. In summary, the inhibitor experiments conducted show that HO-1 is involved in the proautophagic effects of CBD on HUVEC and thus promotes the survival of these cells. However, excessive HO-1 expression by treatment with 10 µM CBD induces HUVEC apoptosis.

HO-1-dependent autophagy has been demonstrated in different cell types [13,14,15,16]. In this context, any of the catabolic end products of the HO-1 reaction (i.e., CO, biliverdine/bilirubin, iron ions) released by heme degradation could be involved in the induction of autophagy. Studies on the role of HO-1 products in the autophagy process showed that CO mediates corresponding proautophagic effects [55,56]. While a low CO concentration inhibits glycolysis in endothelial cells and stimulates ATP release by oxidative phosphorylation in conjunction with an increased tricarboxylic acid (TCA) cycle, a high CO concentration inhibits the respiratory chain at the level of cytochrome oxidase [57,58]. The fact that WST-1 is primarily reduced by TCA cycle-derived NADH [59] supports a possible role of low HO-1-dependent CO concentrations in increasing metabolic activity, which was observed after the incubation of HUVEC with 6 µM CBD. Indeed, the inhibitory effect of the autophagy inhibitor bafilomycin A_1_ on mitochondrial activity increased by 6 µM CBD underlines the importance of HO-1-mediated autophagy for the metabolic activity and survival of HUVEC. Since autophagy is an adaptive response to the increased metabolic demand, it can be deduced that autophagy in the presence of CO preferentially provides substrates for the TCA cycle, which maintains mitochondrial energy metabolism [60,61]. In contrast, it is assumed that it is the increased CO concentrations resulting from strong HO-1 expression (e.g., triggered by 10 µM CBD) that reduce mitochondrial activity and induce apoptosis. Regarding the dual role of HO-1 for cellular viability, it has also been suggested to use the CRISPR/dCas9 system to activate cellular HO-1 expression to an optimal level, which for example guarantees the survival of transplanted stem cells in patients with ischemic heart disease without triggering the cytotoxic effects associated with excessive HO-1 expression [62].

In connection with autophagy, it should be noted that the latter is not only involved in the regulation of the survival or death of endothelial cells [63,64] but also in the modulation of other important functions of endothelial cells, such as nitric oxide production [65], hemostasis/thrombosis [66], and angiogenesis [52]. Pro-angiogenic effects of autophagy have been observed in human endothelial cells treated with adipokine chemerin [67]. For the phytocannabinoid investigated in the present study, another work of our group demonstrated a direct pro-angiogenic effect of 3 µM CBD at the level of migration and tube formation of HUVEC [31], suggesting a possible link to the autophagy shown here. On the other hand, it was also reported that the induction of autophagy by inhibition of mechanistic target of rapamycin (mTOR) with rapamycin reduces the regenerative and angiogenic capacities of endothelial cells both in vitro and in vivo [68].

For CBD, there are conflicting data on the influence on the cellular redox status. A protective effect of CBD is supported by several studies that have demonstrated a reduction of oxidative stress by CBD in endothelial cells, cardiomyocytes, and in a variety of other cell types and animal models of inflammation [25,26,69,70,71]. On the other hand, our results show a ROS-dependent pro-oxidative effect of CBD as the basis of increased HO-1 expression, the latter leading to autophagy and apoptosis of HUVEC, depending on CBD concentration. In accordance with our results, the reports by other authors also showed ROS-dependent apoptosis induction by CBD in leukemia cells, thymocytes, lymphocytes, splenocytes, and monocytes [72,73,74,75,76]. In a study on oligodendrocyte progenitor cells, CBD at 1 µM protected the cells from hydrogen peroxide and lipopolysaccharide/interferon-γ-induced death by reducing ROS production and stress of the endoplasmic reticulum, while the treatment of cells with higher CBD concentrations (2.5 µM and 5 µM) without further stressors induced cytotoxic effects [77]. In addition, a sensitizing/enhancing effect on the toxic effect of redox-active toxins such as hydrogen peroxide or 6-hydroxydopamine on CBD could be demonstrated during neuronal differentiation of cells [78]. Finally, a study using mouse hepatocytes and electron spin resonance spectroscopy demonstrated the generation of ROS by the metabolism of the CBD [79].

Clearly, more research is needed to understand the complex, most likely bidirectional interaction between the CBD-induced Nrf2/HO-1 upregulation and the oxidatively driven proautophagic/proapoptotic cellular process. In this context, it was surprising that the Nrf2 induction registered in the presence of the proapoptotic CBD concentration (10 µM) was lower than that of 6 µM CBD, although 10 µM CBD produced a comparatively stronger HO-1 induction. One reason for this apparent contradiction could be a recently described apoptosis-related inactivation of Nrf2 [80]. In the corresponding study, curcumin initiated an induction of Nrf2 but caused a p53-independent reduction of total and nuclear Nrf2 protein levels at later times of activation [80]. In line with this, earlier studies had already shown that Nrf2 can also be degraded in oxidatively stressed cells, whereby a Keap1-independent process in the cell nucleus mediated by the redox-insensitive Neh6 degron is initiated [81].

Furthermore, the exact, obviously cell type-dependent mechanism of HO-1 induction by CBD poses a challenge for future investigations. In this context, a recently published study showed that CBD mediates HO-1 expression in keratinocytes Nrf2 independently by inducing the cytosolic proteasomal degradation of the transcriptional repressor BTB and CNC homology 1 (BACH1) and its nuclear export [43]. Accordingly, no inhibition of the CBD-induced HO-1 expression in the presence of Nrf2 siRNA could be registered in the respective work [43]. Although in our hands the essential role of Nrf2 in HO-1 expression was underlined by a significant inhibition of CBD-induced HO-1 expression by Nrf2 siRNA, the partial inhibition achieved under these experimental conditions cannot, however, exclude an additional involvement of BACH1 regulation.

Finally, it remains to be clarified whether the increase in metabolic activity caused by 6 µM CBD is causally related to CBD-induced HO-1 expression. Such a relationship is supported by inhibitor experiments with SnPPIX, which show an approximately 43% reduction of this effect, although this inhibition could not be substantially confirmed by Nrf2 siRNA (approximately 17% reduction) and not at all by NAC.

Lastly, it should be mentioned that the apoptosis of HUVEC induced in our hands by 10 µM CBD is consistent with the results describing an anti-angiogenic effect of HUVEC by CBD concentrations of ≥9 µM [82]. However, in the latter study, it was found that cytostasis, but not the induction of apoptosis, was responsible for the observed decrease in metabolic activity. The reasons for these discrepant findings are unclear, but they could be due to different culture conditions. While the culture medium we used contained ECGS, FCS, EGF, bFGF, heparin, and hydrocortisone, Solinas et al. [82] applied a HUVEC medium, which included FCS and vascular endothelial growth factor. Against this background, different supplements that may lead to phenotypic heterogeneity [83] could explain the different sensitivity of HUVEC to high CBD concentrations in the different studies.

## 5. Conclusions

In summary, the present study demonstrates that the non-psychoactive cannabinoid CBD promotes ROS-dependent HO-1 expression in endothelial cells, followed by HO-1-dependent protective autophagy (Figure 8). This protection is maintained up to a certain CBD concentration (up to 6 µM in the present study), but it is then no longer sufficient to protect the cells from the likewise HO-1-dependent apoptotic cell death. HO-1 thereby represents a dual critical modulator of apoptosis. The data presented here show for the first time a functional effect of cannabinoid-induced HO-1 on endothelial viability and thus provide new impulses for the investigation of CBD-based therapeutic strategies in cardiovascular diseases and of possible limitations in the application of higher doses.

## Figures and Tables

**Table 1:** Impact of CuPPIX, a non-HO-1-inhibiting structural analogue of SnPPIX, on CBD-induced changes of viability, HO-1 expression, autophagy, and apoptosis of HUVEC. The cells were pre-incubated with 25 µM CuPPIX for 30 min and then further co-incubated with 6 or 10 µM CBD for another 24 h. Expression values were normalized to β-actin. Percent control represents comparison with vehicle-treated cells (100%) in the absence of test substance. Values are means ± SEM of n = 3–4 (expression data) or n = 8 experiments (metabolic activity). Expression values were obtained from densitometric analysis of blots. * *p* < 0.05 vs. corresponding vehicle control, # *p* < 0.05 vs. CBD-treated group; one-way ANOVA with Bonferroni´s post hoc test.

Treatment Group	Metabolic Activity (%)	HO-1 Expression (%)	LC3A/B-I Expression (%)	LC3A/B-II Expression (%)	Caspase-3 (17 kDa) Expression (%)
Vehicle	100.0 ± 6.7	100.0 ± 8.3	100.0 ± 20.7	100.0 ± 19.2	100.0 ± 39.2
6 µM CBD	120.6 ± 7.3	282.3 ± 28.8*	143.1 ± 9.9	191.7 ± 11.9	83.5 ± 13.7
CuPPIX + CBD	132.0 ± 7.5	334.2 ± 37.2	316.0 ± 45.1#	386.8 ± 53.1#	238.8 ± 46.0
CuPPIX	111.9 ± 5.8	168.9 ± 17.0	190.4 ± 24.8	198.1 ± 31.3	160.0 ± 50.5
Vehicle	100.0 ± 4.5	100.0 ± 17.4	100.0 ± 9.0	100.0 ± 9.4	100.0 ± 5.1
10 µM CBD	51.6 ± 5.3*	3683.2 ± 978.0	106.5 ± 21.3	424.7 ± 85.7*	256.9 ± 17.5*
CuPPIX + CBD	69.3 ± 5.1	5755.6 ± 1525.5	157.6 ± 8.3	615.3 ± 88.0#	333.4 ± 54.7
CuPPIX	108.7 ± 5.9	300.0 ± 69.5	188.6 ± 38.7	204.7 ± 14.3	142.1 ± 2.6

## References

[1] Lusis A.J. (2000). Atherosclerosis. Nature.

[2] Singh R.B., Mengi S.A., Xu Y.J., Arneja A.S., Dhalla N.S. (2002). Pathogenesis of atherosclerosis: A multifactorial process. Exp. Clin. Cardiol..

[3] Li H., Horke S., Förstermann U. (2014). Vascular oxidative stress, nitric oxide and atherosclerosis. Atherosclerosis.

[4] Yang X., Li Y., Li Y., Ren X., Zhang X., Hu D., Gao Y., Xing Y., Shang H. (2017). Oxidative stress-mediated atherosclerosis: Mechanisms and therapies. Front. Physiol..

[5] Dimmeler S., Zeiher A.M. (2000). Endothelial cell apoptosis in angiogenesis and vessel regression. Circ. Res..

[6] Rössig L., Dimmeler S., Zeiher A.M. (2001). Apoptosis in the vascular wall and atherosclerosis. Basic Res. Cardiol..

[7] Li J., Xiong J., Yang B., Zhou Q., Wu Y., Luo H., Zhou H., Liu N., Li Y., Song Z. (2015). Endothelial cell apoptosis induces TGF-β signaling-dependent host endothelial-mesenchymal transition to promote transplant arteriosclerosis. Am. J. Transplant..

[8] Paine A., Eiz-Vesper B., Blasczyk R., Immenschuh S. (2010). Signaling to heme oxygenase-1 and its anti-inflammatory therapeutic potential. Biochem. Pharmacol..

[9] Brouard S., Otterbein L.E., Anrather J., Tobiasch E., Bach F.H., Choi A.M., Soares M.P. (2000). Carbon monoxide generated by heme oxygenase 1 suppresses endothelial cell apoptosis. J. Exp. Med..

[10] Kushida T., Li Volti G., Quan S., Goodman A., Abraham N.G. (2002). Role of human heme oxygenase-1 in attenuating TNF-alpha-mediated inflammation injury in endothelial cells. J. Cell Biochem..

[11] Asija A., Peterson S.J., Stec D.E., Abraham N.G. (2007). Targeting endothelial cells with heme oxygenase-1 gene using VE-cadherin promoter attenuates hyperglycemia-mediated cell injury and apoptosis. Antioxid. Redox Signal..

[12] Castilho Á., Aveleira C.A., Leal E.C., Simões N.F., Fernandes C.R., Meirinhos R.I., Baptista F.I., Ambrósio A.F. (2012). Heme oxygenase-1 protects retinal endothelial cells against high glucose- and oxidative/nitrosative stress-induced toxicity. PLoS ONE.

[13] Waltz P., Carchman E.H., Young A.C., Rao J., Rosengart M.R., Kaczorowski D., Zuckerbraun B.S. (2011). Lipopolysaccaride induces autophagic signaling in macrophages via a TLR4, heme oxygenase-1 dependent pathway. Autophagy.

[14] Lin T.K., Chen S.D., Chuang Y.C., Lin H.Y., Huang C.R., Chuang J.H., Wang P.W., Huang S.T., Tiao M.M., Chen J.B. (2014). Resveratrol partially prevents rotenone-induced neurotoxicity in dopaminergic SH-SY5Y cells through induction of heme oxygenase-1 dependent autophagy. Int. J. Mol. Sci..

[15] Surolia R., Karki S., Kim H., Yu Z., Kulkarni T., Mirov S.B., Carter A.B., Rowe S.M., Matalon S., Thannickal V.J. (2015). Heme oxygenase-1-mediated autophagy protects against pulmonary endothelial cell death and development of emphysema in cadmium-treated mice. Am. J. Physiol. Lung Cell Mol. Physiol..

[16] Zou S., Sun H., Candiotti K.A., Peng Y., Zhang Q., Xiao W., Zhao S., Wu L., Yang J. (2018). Octreotide protects against hepatic ischemia/reperfusion injury via HO-1-mediated autophagy. Acta Biochim. Biophys. Sin. (Shanghai).

[17] Stocker R., Perrella M.A. (2006). Heme oxygenase-1: A novel drug target for atherosclerotic diseases?. Circulation.

[18] Chen Y.H., Kuo K.L., Hung S.C., Hsu C.C., Chen Y.H., Tarng D.C. (2014). Length polymorphism in heme oxygenase-1 and risk of CKD among patients with coronary artery disease. J. Am. Soc. Nephrol..

[19] Chen S., Khan Z.A., Barbin Y., Chakrabarti S. (2004). Pro-oxidant role of heme oxygenase in mediating glucose-induced endothelial cell damage. Free Radic. Res..

[20] Yang C.M., Lin C.C., Hsieh H.L. (2017). High-Glucose-Derived Oxidative Stress-Dependent Heme Oxygenase-1 Expression from Astrocytes Contributes to the Neuronal Apoptosis. Mol. Neurobiol..

[21] Suttner D.M., Dennery P.A. (1999). Reversal of HO-1 related cytoprotection with increased expression is due to reactive iron. FASEB J..

[22] Maruhashi K., Kasahara Y., Ohta K., Wada T., Ohta K., Nakamura N., Toma T., Koizumi S., Yachie A. (2004). Paradoxical enhancement of oxidative cell injury by overexpression of heme oxygenase-1 in an anchorage-dependent cell ECV304. J. Cell Biochem..

[23] D’Amico G., Lam F., Hagen T., Moncada S. (2006). Inhibition of cellular respiration by endogenously produced carbon monoxide. J. Cell Sci..

[24] Zuckerbraun B.S., Chin B.Y., Bilban M., d’Avila J.C., Rao J., Billiar T.R., Otterbein L.E. (2007). Carbon monoxide signals via inhibition of cytochrome c oxidase and generation of mitochondrial reactive oxygen species. FASEB J..

[25] Rajesh M., Mukhopadhyay P., Bátkai S., Haskó G., Liaudet L., Drel V.R., Obrosova I.G., Pacher P. (2007). Cannabidiol attenuates high glucose-induced endothelial cell inflammatory response and barrier disruption. Am. J. Physiol. Heart Circ. Physiol..

[26] Rajesh M., Mukhopadhyay P., Bátkai S., Patel V., Saito K., Matsumoto S., Kashiwaya Y., Horváth B., Mukhopadhyay B., Becker L. (2010). Cannabidiol attenuates cardiac dysfunction, oxidative stress, fibrosis, and inflammatory and cell death signaling pathways in diabetic cardiomyopathy. J. Am. Coll. Cardiol..

[27] Mechoulam R., Peters M., Murillo-Rodriguez E., Hanus L.O. (2007). Cannabidiol—Recent advances. Chem. Biodivers..

[28] Jacobsson S.O., Wallin T., Fowler C.J. (2001). Inhibition of rat C6 glioma cell proliferation by endogenous and synthetic cannabinoids. Relative involvement of cannabinoid and vanilloid receptors. J. Pharmacol. Exp. Ther..

[29] Mukherjee S., Adams M., Whiteaker K., Daza A., Kage K., Cassar S., Meyer M., Yao B.B. (2004). Species comparison and pharmacological characterization of rat and human CB_2_ cannabinoid receptors. Eur. J. Pharmacol..

[30] Ramer R., Hinz B. (2008). Inhibition of cancer cell invasion by cannabinoids via increased expression of tissue inhibitor of matrix metalloproteinases-1. J. Natl. Cancer Inst..

[31] Ramer R., Fischer S., Haustein M., Manda K., Hinz B. (2014). Cannabinoids inhibit angiogenic capacities of endothelial cells via release of tissue inhibitor of matrix metalloproteinases-1 from lung cancer cells. Biochem. Pharmacol..

[32] Nguyen T., Sherratt P.J., Huang H.C., Yang C.S., Pickett C.B. (2003). Increased protein stability as a mechanism that enhances Nrf2-mediated transcriptional activation of the antioxidant response element. Degradation of Nrf2 by the 26 S proteasome. J. Biol. Chem..

[33] Na H.K., Surh Y.J. (2014). Oncogenic potential of Nrf2 and its principal target protein heme oxygenase-1. Free Radic. Biol. Med..

[34] Kabeya Y., Mizushima N., Ueno T., Yamamoto A., Kirisako T., Noda T., Kominami E., Ohsumi Y., Yoshimori T. (2000). LC3, a mammalian homologue of yeast Apg8p, is localized in autophagosome membranes after processing. EMBO J..

[35] Barth S., Glick D., Macleod K.F. (2010). Autophagy: Assays and artifacts. J. Pathol..

[36] Kimura S., Fujita N., Noda T., Yoshimori T. (2009). Monitoring autophagy in mammalian cultured cells through the dynamics of LC3. Methods Enzymol..

[37] Oh C.J., Park S., Kim J.Y., Kim H.J., Jeoung N.H., Choi Y.K., Go Y., Park K.G., Lee I.K. (2014). Dimethylfumarate attenuates restenosis after acute vascular injury by cell-specific and Nrf2-dependent mechanisms. Redox Biol..

[38] Kim S.J., Park C., Lee J.N., Lim H., Hong G.Y., Moon S.K., Lim D.J., Choe S.K., Park R. (2015). Erdosteine protects HEI-OC1 auditory cells from cisplatin toxicity through suppression of inflammatory cytokines and induction of Nrf2 target proteins. Toxicol. Appl. Pharmacol..

[39] Tebay L.E., Robertson H., Durant S.T., Vitale S.R., Penning T.M., Dinkova-Kostova A.T., Hayes J.D. (2015). Mechanisms of activation of the transcription factor Nrf2 by redox stressors, nutrient cues, and energy status and the pathways through which it attenuates degenerative disease. Free Radic. Biol. Med..

[40] Schwartz M., Böckmann S., Hinz B. (2018). Up-regulation of heme oxygenase-1 expression and inhibition of disease-associated features by cannabidiol in vascular smooth muscle cells. Oncotarget.

[41] Juknat A., Pietr M., Kozela E., Rimmerman N., Levy R., Coppola G., Geschwind D., Vogel Z. (2012). Differential transcriptional profiles mediated by exposure to the cannabinoids cannabidiol and Δ9-tetrahydrocannabinol in BV-2 microglial cells. Br. J. Pharmacol..

[42] Juknat A., Pietr M., Kozela E., Rimmerman N., Levy R., Gao F., Coppola G., Geschwind D., Vogel Z. (2013). Microarray and pathway analysis reveal distinct mechanisms underlying cannabinoid-mediated modulation of LPS-induced activation of BV-2 microglial cells. PLoS ONE.

[43] Casares L., García V., Garrido-Rodríguez M., Millán E., Collado J.A., García-Martín A., Peñarando J., Calzado M.A., de la Vega L., Muñoz E. (2020). Cannabidiol induces antioxidant pathways in keratinocytes by targeting BACH1. Redox Biol..

[44] Louvet A., Teixeira-Clerc F., Chobert M.N., Deveaux V., Pavoine C., Zimmer A., Pecker F., Mallat A., Lotersztajn S. (2011). Cannabinoid CB_2_ receptors protect against alcoholic liver disease by regulating Kupffer cell polarization in mice. Hepatology.

[45] Steib C.J., Gmelin L., Pfeiler S., Schewe J., Brand S., Göke B., Gerbes A.L. (2013). Functional relevance of the cannabinoid receptor 2—heme oxygenase pathway: A novel target for the attenuation of portal hypertension. Life Sci..

[46] Wang Y., Ma S., Wang Q., Hu W., Wang D., Li X., Su T., Qin X., Zhang X., Ma K. (2014). Effects of cannabinoid receptor type 2 on endogenous myocardial regeneration by activating cardiac progenitor cells in mouse infarcted heart. Sci. China Life Sci..

[47] Blázquez C., González-Feria L., Alvarez L., Haro A., Casanova M.L., Guzmán M. (2004). Cannabinoids inhibit the vascular endothelial growth factor pathway in gliomas. Cancer Res..

[48] Li H., Wood J.T., Whitten K.M., Vadivel S.K., Seng S., Makriyannis A., Avraham H.K. (2013). Inhibition of fatty acid amide hydrolase activates Nrf2 signalling and induces heme oxygenase 1 transcription in breast cancer cells. Br. J. Pharmacol..

[49] Wang Q., Liang B., Shirwany N.A., Zou M.H. (2011). 2-Deoxy-D-glucose treatment of endothelial cells induces autophagy by reactive oxygen species-mediated activation of the AMP-activated protein kinase. PLoS ONE.

[50] Teng R.J., Du J., Welak S., Guan T., Eis A., Shi Y., Konduri G.G. (2012). Cross talk between NADPH oxidase and autophagy in pulmonary artery endothelial cells with intrauterine persistent pulmonary hypertension. Am. J. Physiol. Lung Cell Mol. Physiol..

[51] Kroemer G., Mariño G., Levine B. (2010). Autophagy and the integrated stress response. Mol. Cell.

[52] Jiang F. (2016). Autophagy in vascular endothelial cells. Clin. Exp. Pharmacol. Physiol..

[53] Sardana M.K., Kappas A. (1987). Dual control mechanism for heme oxygenase: Tin(IV)- protoporphyrin potently inhibits enzyme activity while markedly increasing content of enzyme protein in liver. Proc. Natl. Acad. Sci. USA.

[54] Chang T., Wu L., Wang R. (2008). Inhibition of vascular smooth muscle cell proliferation by chronic hemin treatment. Am. J. Physiol. Heart Circ. Physiol..

[55] Lee S.J., Ryter S.W., Xu J.F., Nakahira K., Kim H.P., Choi A.M., Kim Y.S. (2011). Carbon monoxide activates autophagy via mitochondrial reactive oxygen species formation. Am. J. Respir. Cell Mol. Biol..

[56] Constantin M., Choi A.J., Cloonan S.M., Ryter S.W. (2012). Therapeutic potential of heme oxygenase-1/carbon monoxide in lung disease. Int. J. Hypertens..

[57] Choi Y.K., Por E.D., Kwon Y.G., Kim Y.M. (2012). Regulation of ROS production and vascular function by carbon monoxide. Oxid. Med. Cell Longev..

[58] Kaczara P., Motterlini R., Kus K., Zakrzewska A., Abramov A.Y., Chlopicki S. (2016). Carbon monoxide shifts energetic metabolism from glycolysis to oxidative phosphorylation in endothelial cells. FEBS Lett..

[59] Berridge M.V., Herst P.M., Tan A.S. (2005). Tetrazolium dyes as tools in cell biology: New insights into their cellular reduction. Biotechnol. Annu. Rev..

[60] Kimura T., Takahashi A., Takabatake Y., Namba T., Yamamoto T., Kaimori J.Y., Matsui I., Kitamura H., Niimura F., Matsusaka T. (2013). Autophagy protects kidney proximal tubule epithelial cells from mitochondrial metabolic stress. Autophagy.

[61] Guo J.Y., Teng X., Laddha S.V., Ma S., Van Nostrand S.C., Yang Y., Khor S., Chan C.S., Rabinowitz J.D., White E. (2016). Autophagy provides metabolic substrates to maintain energy charge and nucleotide pools in Ras-driven lung cancer cells. Genes Dev..

[62] Pan A., Weintraub N.L., Tang Y. (2014). Enhancing stem cell survival in an ischemic heart by CRISPR-dCas9-based gene regulation. Med. Hypotheses.

[63] Uberti F., Lattuada D., Morsanuto V., Nava U., Bolis G., Vacca G., Squarzanti D.F., Cisari C., Molinari C. (2014). Vitamin D protects human endothelial cells from oxidative stress through the autophagic and survival pathways. J. Clin. Endocrinol. Metab..

[64] Dong G., Yang S., Cao X., Yu N., Yu J., Qu X. (2017). Low shear stress-induced autophagy alleviates cell apoptosis in HUVECs. Mol. Med. Rep..

[65] Bharath L.P., Cho J.M., Park S.K., Ruan T., Li Y., Mueller R., Bean T., Reese V., Richardson R.S., Cai J. (2017). Endothelial Cell Autophagy Maintains Shear Stress-Induced Nitric Oxide Generation via Glycolysis-Dependent Purinergic Signaling to Endothelial Nitric Oxide Synthase. Arterioscler. Thromb. Vasc. Biol..

[66] Yau J.W., Singh K.K., Hou Y., Lei X., Ramadan A., Quan A., Teoh H., Kuebler W.M., Al-Omran M., Yanagawa B. (2017). Endothelial-specific deletion of autophagy-related 7 (ATG7) attenuates arterial thrombosis in mice. J. Thorac. Cardiovasc. Surg..

[67] Shen W., Tian C., Chen H. (2013). Oxidative stress mediates chemerin-induced autophagy in endothelial cells. Free Radic. Biol. Med..

[68] Hayashi S., Yamamoto A., You F. (2009). The stent-eluting drugs sirolimus and paclitaxel suppress healing of the endothelium by induction of autophagy. Am. J. Pathol..

[69] Borrelli F., Aviello G., Romano B., Orlando P., Capasso R., Maiello F., Guadagno F., Petrosino S., Capasso F., Di Marzo V. (2009). Cannabidiol, a safe and non-psychotropic ingredient of the marijuana plant Cannabis sativa, is protective in a murine model of colitis. J. Mol. Med. (Berl.).

[70] Pan H., Mukhopadhyay P., Rajesh M., Patel V., Mukhopadhyay B., Gao B., Haskó G., Pacher P. (2009). Cannabidiol attenuates cisplatin-induced nephrotoxicity by decreasing oxidative/nitrosative stress, inflammation, and cell death. J. Pharmacol. Exp. Ther..

[71] Fernández-Ruiz J., Sagredo O., Pazos M.R., García C., Pertwee R., Mechoulam R., Martínez-Orgado J. (2013). Cannabidiol for neurodegenerative disorders: Important new clinical applications for this phytocannabinoid?. Br. J. Clin. Pharmacol..

[72] McKallip R.J., Jia W., Schlomer J., Warren J.W., Nagarkatti P.S., Nagarkatti M. (2006). Cannabidiol-induced apoptosis in human leukemia cells: A novel role of Cannabidiol in the regulation of p22phox and NOX4 expression. Mol. Pharmacol..

[73] Lee C.Y., Wey S.P., Liao M.H., Hsu W.L., Wu H.Y., Jan T.R. (2008). A comparative study on cannabidiol-induced apoptosis in murine thymocytes and EL-4 thymoma cells. Int. Immunopharmacol..

[74] Wu H.Y., Chu R.M., Wang C.C., Lee C.Y., Lin S.H., Jan T.R. (2008). Cannabidiol-induced apoptosis in primary lymphocytes is associated with oxidative stress-dependent activation of caspase-8. Toxicol. Appl. Pharmacol..

[75] Wu H.Y., Jan T.R. (2010). Cannabidiol hydroxyquinone-induced apoptosis of splenocytes is mediated predominantly by thiol depletion. Toxicol. Lett..

[76] Wu H.Y., Huang C.H., Lin Y.H., Wang C.C., Jan T.R. (2018). Cannabidiol induced apoptosis in human monocytes through mitochondrial permeability transition pore-mediated ROS production. Free Radic. Biol. Med..

[77] Mecha M., Torrao A.S., Mestre L., Carrillo-Salinas F.J., Mechoulam R., Guaza C. (2012). Cannabidiol protects oligodendrocyte progenitor cells from inflammation-induced apoptosis by attenuating endoplasmic reticulum stress. Cell Death Dis..

[78] Schönhofen P., de Medeiros L.M., Bristot I.J., Lopes F.M., De Bastiani M.A., Kapczinski F., Crippa J.A., Castro M.A., Parsons R.B., Klamt F. (2015). Cannabidiol exposure during neuronal differentiation sensitizes cells against redox-active neurotoxins. Mol. Neurobiol..

[79] Usami N., Yamamoto I., Watanabe K. (2008). Generation of reactive oxygen species during mouse hepatic microsomal metabolism of cannabidiol and cannabidiol hydroxy-quinone. Life Sci..

[80] Méndez-García L.A., Martínez-Castillo M., Villegas-Sepúlveda N., Orozco L., Córdova E.J. (2019). Curcumin induces p53-independent inactivation of Nrf2 during oxidative stress-induced apoptosis. Hum. Exp. Toxicol..

[81] McMahon M., Thomas N., Itoh K., Yamamoto M., Hayes J.D. (2004). Redox-regulated turnover of Nrf2 is determined by at least two separate protein domains, the redox-sensitive Neh2 degron and the redox-insensitive Neh6 degron. J. Biol. Chem..

[82] Solinas M., Massi P., Cantelmo A.R., Cattaneo M.G., Cammarota R., Bartolini D., Cinquina V., Valenti M., Vicentini L.M., Noonan D.M. (2012). Cannabidiol inhibits angiogenesis by multiple mechanisms. Br. J. Pharmacol..

[83] Aird W.C. (2012). Endothelial cell heterogeneity. Cold Spring Harb. Perspect. Med..

